# Reversal of the CD8^+^ T-Cell Exhaustion Induced by Chronic HIV-1 Infection Through Combined Blockade of the Adenosine and PD-1 Pathways

**DOI:** 10.3389/fimmu.2021.687296

**Published:** 2021-06-10

**Authors:** Jing Li, Hui-Huang Huang, Bo Tu, Ming-Ju Zhou, Wei Hu, Yu-Long Fu, Xiao-Yu Li, Tao Yang, Jin-Wen Song, Xing Fan, Yan-Mei Jiao, Ruo-Nan Xu, Ji-Yuan Zhang, Chun-Bao Zhou, Jin-Hong Yuan, Cheng Zhen, Ming Shi, Fu-Sheng Wang, Chao Zhang

**Affiliations:** ^1^Peking University 302 Clinical Medical School, Beijing, China; ^2^Department of Infectious Diseases, The Fifth Medical Center of Chinese PLA General Hospital, National Clinical Research Center for Infectious Diseases, Beijing, China; ^3^Medical School of Chinese PLA, Beijing, China; ^4^Savaid Medical School, University of Chinese Academy of Sciences, Beijing, China; ^5^Bengbu Medical University, Bengbu, China

**Keywords:** PD-1^+^CD39^+^ CD8^+^ T cells, HIV-1, cytotoxic T cells, PD-1/PD-L1, CD39/adenosine pathway

## Abstract

**Background:**

Targeting immune checkpoints for HIV treatment potentially provides a double benefit resulting from the ability to restore viral-specific CD8^+^ T-cell functions and enhance HIV production from reservoir cells. Despite promising pre-clinical data, PD-1 blockade alone in HIV-1-infected patients with advanced cancer has shown limited benefits in controlling HIV, suggesting the need for additional targets beyond PD-1. CD39 and PD-1 are highly co-expressed on CD8^+^ T cells in HIV-1 infection. However, the characteristics of CD39 and PD-1 dual-positive CD8^+^ T-cell subsets in chronic HIV-1 infection remain poorly understood.

**Methods:**

This study enrolled 72 HIV-1-infected patients, including 40 treatment naïve and 32 ART patients. A total of 11 healthy individuals were included as controls. Different subsets of CD8^+^ T cells defined by CD39 and/or PD-1 expression were studied by flow cytometry. The relationships between the frequencies of the different subsets and parameters indicating HIV-1 disease progression were analyzed. Functional (i.e., cytokine secretion, viral inhibition) assays were performed to evaluate the impact of the blockade of adenosine and/or PD-1 signaling on CD8^+^ T cells.

**Results:**

The proportions of PD-1^+^, CD39^+^, and PD-1^+^CD39^+^ CD8^+^ T cells were significantly increased in treatment naïve patients but were partially lowered in patients on antiretroviral therapy. In treatment naïve patients, the proportions of PD-1^+^CD39^+^ CD8^+^ T cells were negatively correlated with CD4^+^ T-cell counts and the CD4/CD8 ratio, and were positively correlated with viral load. CD39^+^CD8^+^ T cells expressed high levels of the A2A adenosine receptor and were more sensitive to 2-chloroadenosine-mediated functional inhibition than their CD39^-^ counterparts. *In vitro*, a combination of blocking CD39/adenosine and PD-1 signaling showed a synergic effect in restoring CD8^+^ T-cell function, as evidenced by enhanced abilities to secrete functional cytokines and to kill autologous reservoir cells.

**Conclusion:**

In patients with chronic HIV-1 infection there are increased frequencies of PD-1^+^, CD39^+^, and PD-1^+^CD39^+^ CD8^+^ T cells. In treatment naïve patients, the frequencies of PD-1^+^CD39^+^ CD8^+^ T cells are negatively correlated with CD4^+^ T-cell counts and the CD4/CD8 ratio and positively correlated with viral load. Combined blockade of CD39/adenosine and PD-1 signaling *in vitro* may exert a synergistic effect in restoring CD8^+^ T-cell function in HIV-1-infected patients.

## Introduction

CD8^+^ T cells are a critical component of the cellular immune response to viral infections. A non-redundant role for CD8^+^ T cells in controlling HIV-1 infections is well recognized, particularly in HIV controllers, as there is a very small proportion of people living with HIV (PLWH) who manage to spontaneously control viral replication and maintain stable CD4^+^ T-cell counts without the need for antiretroviral therapy (ART) (1, 2). However, for the vast majority of PLWH, persistent viral infection induces a dysfunction in CD8^+^ T cells. The virus has adopted numerous strategies to evade recognition and killing by CD8^+^ T cells, such as impairing T helper function due to a profound depletion of CD4^+^ T cells (3), mutational escape (4), and impairing antigen-presenting by down-regulating MHC-I molecules on the surface of infected cells (5). At the same time, by altering the production of cytokines and the engagement of receptors, the HIV-1 virus impairs CD8^+^ T-cell signaling, resulting in inappropriate T-cell receptor (TCR) stimulation, which leads to T-cell exhaustion (6). Therefore, targeted therapies that can restore the function of CD8^+^ T cells represent an important strategy for HIV treatment.

The advent of ARTs has revolutionized the treatment of PLWH, with many expected to have a nearly normal life expectancy (7). However, due to the persistence of the viral reservoir, any interruption in ARTs inevitably leads to a rapid rebound of viremia, hence life-long treatment is required. Immune checkpoint inhibitors (ICIs) have shown success in the treatment of various cancers, however ICI treatment for HIV-1 infection is less studied. Pre-clinical studies have shown that blockade of programmed cell death protein 1 (PD-1) enhances T-cell function during Simian immunodeficiency virus (SIV) infection (8–12), and the combination of an anti-PD-1 antibody with ART provides therapeutic benefit against SIV (13). *In vitro* studies have also demonstrated that the administration of ICI to PBMCs from PLWH who are receiving ART can promote a reversal of latent reservoir and make it easier for the virus to be recognized by immune cells (8). These advances provide a strong rationale for ICI based clinical trials in PLWH.

Currently, ICI therapy studies for PLWH are mostly restricted to patients with advanced cancer (14). Based on the published data from several evaluable ICI trials that allow the enrollment of PLWH, the feasibility, safety, and efficacy of PD-1 blockade of tumors in PLWH are similar to those observed in patients without HIV (15–17). Remarkably, researchers found that there was a drastic decrease in the HIV reservoir in a patient with lung cancer who had been treated with nivolumab (18). This finding suggests that prospective clinical trials that focus exclusively on PLWH could shed light on the feasibility of this therapeutic approach. Unfortunately, the clinical benefits of HIV control in response to ICI treatment were not consistently observed in many other patients with PLWH who also have cancer (19). These inconsistencies may be due to the inefficiency of targeting PD-1 alone and poor tolerance for the early generation of ICI drugs (20). Seeking more effective ICI(s) is therefore a promising approach for the treatment of HIV.

CD39 is an ectonucleotidase that converts pro-inflammatory ATP signals into AMP and then in connection with another ectoenzyme CD73, AMP is converted into immunosuppressive adenosine (21). Studies have reported that the extracellular adenosine pathway is related to the progression of AIDS (22, 23). Compared with healthy subjects, T cells from patients with PLWH have higher expression levels of A2AR and higher intracellular cAMP levels. In CD39^+^ Tregs, IL-2 production is inhibited *via* the CD39/adenosine/cAMP pathway (24, 25). Moreover, the CD39/adenosine signal also has a potential impact on the function of CD8^+^ T cells in HIV-1 infections. Recent studies have shown that CD39^+^CD8^+^ T cells are characterized by terminal exhaustion, immunoregulatory activity (23, 26–29), implying that this cell population might be useful as a biomarker and therapeutic target for the treatment of advanced tumors and chronic infections. CD39^+^CD8^+^ T cells often co-express PD-1 and are enriched with genes that are hallmarks of T-cell exhaustion. CD39 is preferentially upregulated on virus-specific CD8^+^ T cells with a high antigen burden (23, 27, 30). In addition, studies have shown that HIV-1 infection can induce the proliferation of CD8^+^CD28^-^CD127^lo^CD39^+^ Treg cells, and their frequency is related to the signs of chronic immune cell activation (28). The CD39^+^CD8^+^ T-cell subset is related to the clinical progression of acquired immune deficiency syndrome (AIDS), but the characteristics and clinical significance of this cell subset during chronic HIV-1 infection are not well understood. Blocking the adenosine pathway promotes the recovery of exhausted effector cell function, which may be used as a new potential immune checkpoint to treat HIV-1 infection.

In this study, we evaluated CD39 and PD-1 expression on CD8^+^ T cells in HIV-1 infected individuals at different stages of infection. We found that in patients with HIV-1 infections, there are increased frequencies of PD-1^+^, CD39^+^, and PD-1^+^CD39^+^ CD8^+^ T cells, which are inversely correlated with CD4^+^ T-cell counts and positively correlated with viral load in treatment naïve patients. *In vitro*, combined blockade of PD-1 and adenosine signaling showed a synergic effect in restoring CD8^+^ T-cell function in ART patients. These findings may be leveraged to develop novel checkpoint therapies for HIV-1 infections.

## Materials and Methods

### Subjects

A total of 11 healthy controllers (HCs) and 72 PLWH were enrolled in this study. For PLWH, 40 were treatment naïve patients (TNs, who exhibited a typical progressive disease with peripheral CD4^+^ T-cell counts < 500 cells/µL and plasma viral RNA > 1000 copies/mL), and 32 ART patients (who underwent ART for more than two years with peripheral CD4^+^ T-cell counts above 350 cells/μL and plasma HIV-1 RNA < 80 copies/mL) (**Table 1**). The exclusion criteria included pregnancy, hepatitis B virus infections, hepatitis C virus infections, tuberculosis, Dengue virus infections, and a moribund status. All samples were collected with the Fifth Medical Center of PLA General Hospital Research Ethics Committee’s approval and all individuals were required to provide their written informed consent in accordance with the Declaration of Helsinki.

**Table 1:** Characteristics of patients in this study.

Sample	HCs	TNs	ARTs
Cases (n)	11	40	32
Age (years)	28 (22~34)	37 (21~64)	34 (21~64)
Gender (M/F)	6/5	38/2	32/0
viral load (copies/mL)	NA	152, 420 (4, 039~969, 625)	<LDL
CD4 cell count (cells/μL)	784 (481~1, 105)	351 (57~768)	568 (51~1, 164)
CD8 count (cells/μL)	616 (421~865)	980 (353~1, 954)	1046 (415~3, 082)
CD4/CD8 ratio	1.29 (0.75~1.71)	0.39 (0.16~0.87)	0.63 (0.09~1.69)

Data were expressed as the median (range).

HCs, healthy controllers; TNs, treatment naïve patients; ARTs, patients under antiretroviral therapy; M, male; F, female.

NA, not applicable; LDL, lower detection limit.

### Isolation of PBMCs and Cell Sorting

Peripheral blood mononuclear cells (PBMCs) were isolated from EDTA anti-coagulated venous blood by Ficoll-Hypaque (MD Pacific Biotechnology, Tianjin, China) density gradient centrifugation. CD8^+^ T cells and CD4^+^ T cells were separately isolated by positive selection using the MiniMACS kit (Miltenyi Biotech, Bergisch-Gladbach, Germany) and by negative selection using a magnetic selection kit (StemCell Technologies, Vancouver, Canada) according to the manufacturer’s instructions.

### Flow Cytometry

For phenotypic staining, PBMCs were extracellularly stained using antibodies specific to respective markers, including anti-CD3-APC-Cy7 (BD Biosciences, Franklin Lakes, NJ, USA), anti-CD8-APC-Cy7, anti-CD8-BV510 (BioLegend, San Diego, CA, USA), rabbit anti-human A2AR mAb (Thermo Fisher Scientific, Waltham, MA, USA), Alexa Fluor 488 goat anti-rabbit IgG (Life Technologies, Carlsbad, CA, USA), anti-PD-1-FITC (eBioscience, Waltham, MA, USA), and anti-CD39-PE-Cy7 (BioLegend) for 30 min at room temperature. For detection of antigen specific CD8^+^ T cells, PBMCs from HLA-A*0201 positive TNs were stained with HIV-gag (SLYNTVATL; ProImmune, Oxford, UK) APC-conjugated pentamers before staining with antibodies. The cells were washed with FACS buffer and assessed by flow cytometry. After extracellular staining, the cells were further treated with Cytofix/Cytoperm (BD PharMingen, San Diego, CA, USA) according to the manufacturer’s instructions and were then intracellularly stained with antibodies against IL-2-PE (BioLegend), IFN-γ-APC (BioLegend) and HIV-core Ag (KC57)-FITC (Beckman Coulter, Inc. USA) at 4°C for 30 min. Staining for the intracellular transcription factor TOX was performed using the FOXP3/Transcription Factor Staining kit (eBioscience) and then stained with anti-TOX-APC (eBioscience). Cells were fixed in 0.5% formaldehyde and then analyzed by flow cytometry on a BD Canto II flow cytometer (BD Biosciences). Data were acquired on a BD-FACSCanto (BD Biosciences) and analyzed using FlowJo software V10 (Tree Star Inc., Ashland, OR, USA).

### Intracellular Cytokine Assay

PBMCs were cultured in RPMI 1640 containing 10% fetal bovine serum and then stimulated with anti-CD3 (1 ng/mL) (T&L Biological Technology, Beijing, China) and anti-CD28 (1 ng/mL) (BioLegend) monoclonal antibodies for 6 h or an HIV pol, gag, and env peptide pool (1 μg/mL each) (JPT, Berlin, Germany) for 6h for different experiments. The cells were also treated with or without AZD4635 (0.2 µM) (Selleck Chemicals, Houston, USA) and/or an anti-PD-L1 antibody (10 µg/mL) (eBioscience). For analyses of cytokine production in stimulated cells, GlogiPlug (eBioscience) was added 6h before harvest. The cells were collected and the intracellular expression levels of IL-2 and IFN-γ were measured by flow cytometry, as described above.

### Detection of HIV DNA

Total cellular DNA was extracted from one million frozen PBMCs using Qiagen QIAsymphony DNA Mini Kits (Qiagen, Valencia, CA). HIV DNA was quantified using a fluorescence-based real-time SUPBIO HIV Quantitative Detection Kit (SUPBIO, Guangzhou, China). The quantification range was 10–5 × 10^6^ copies/10^6^ PBMCs.

### Viral Suppression Assay

The viral suppression assay was performed as previously described (31). Sorted CD8^-^ and CD56^-^CD4^+^ T cells were co-cultured in RPMI 1640 medium with 10% FBS, supplemented with phytohemagglutinin (PHA) (5 μg/mL) and IL-2 (500 IU/mL) for 2 days. CD8^+^ T cells were cultured in RPMI 1640 medium supplemented with AZD4635 and/or an anti-PD-L1 antibody. After 2 days of stimulation, the cells were washed two times with RPMI 1640. The activated CD8^-^CD56^-^CD4^+^ T cells and CD8^+^ T cells were co-cultured in RPMI 1640 medium containing IL-2 (500 IU/mL) for 1 day at a ratio of 1:3. Following this, the expression levels of p24 in the CD4^+^ T cells were determined by flow cytometry.

### Statistical Analysis

Statistical analysis was performed using GraphPad Prism software version 8.0 (GraphPad Software, San Diego, CA, USA). Data represent the mean ± SD. Comparisons between two groups were performed using the nonparametric Mann–Whitney U test or a Wilcoxon’s paired test. A paired Student’s t-test was adopted for the analysis of the different treatment groups in **Figures 3** and **4**. Correlations were determined using the Spearman rank correlation test. For all tests, P values < 0.05 indicated a significant difference.

## Results

### The Frequency of PD-1^+^CD39^+^ CD8^+^ T Cells Is Positively Correlated With Viral Load and Negatively Correlated With CD4^+^ T-Cell Counts and the CD4/CD8 Ratio in Patients With Chronic HIV-1 Infection

**Figure 1:**
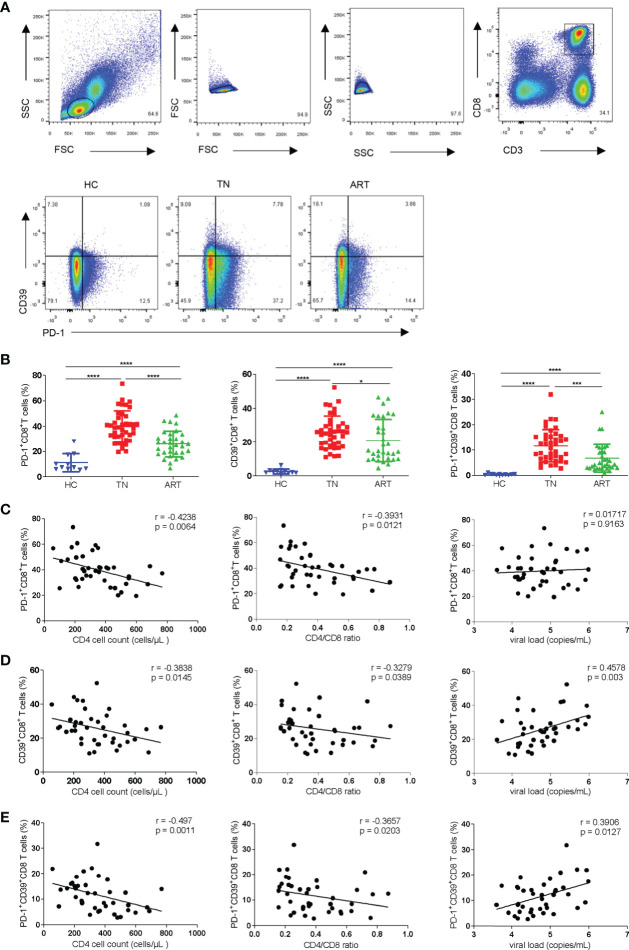
Frequencies of PD-1^+^CD8^+^, CD39^+^CD8^+^, and PD-1^+^CD39^+^CD8^+^ T cells and their correlations with CD4^+^ T-cell counts, the CD4/CD8 ratio, and viral load in HIV-1-infected patients. **(A)** Gating strategy used for the flow cytometry analysis. Representative flow cytometry data for CD39 and PD-1 staining from the peripheral blood of healthy control and HIV-1 infected patients. **(B)** Comparison of the percentages of PD-1^+^CD8^+^ T cells, CD39^+^CD8^+^ T cells and PD-1^+^CD39^+^CD8^+^ T cells in healthy controllers (HCs), treatment naïve patients (TNs), and ART patients (ARTs). **(C–E)** Correlations between the frequencies of PD-1^+^CD8^+^ **(C)**, CD39^+^CD8^+^ **(D)** and PD-1^+^CD39^+^ CD8^+^ **(E)** T cells with CD4^+^ T-cell counts, the CD4/CD8 ratio, and viral load in TNs. Each dot represents one individual. For the statistical analyses, a Mann–Whitney U-test was performed. Correlations were performed using a Spearman rank correlation test. Solid line, linear growth trend; r, correlation coefficient. P values are shown as *p < 0.05, ***p < 0.001, ****p < 0.0001.

To investigate the different subsets of CD8^+^ T cells defined by the expression of PD-1 and/or CD39, we measured the frequencies of PD-1^+^, CD39^+^, and PD-1^+^CD39^+^ CD8^+^ T-cell subsets in the peripheral blood of HCs (n = 11) and PLWH (n = 72; TNs = 40; ARTs = 32) (**Figure 1A**). Compared with HCs, the expression of PD-1^+^, CD39^+^, and PD-1^+^CD39^+^ on CD8^+^T cells in HIV-1 infected patients were all significantly increased, especially in TNs. In ARTs, the proportions of PD-1^+^, CD39^+^, and PD-1^+^CD39^+^ subsets of CD8^+^ T cells were lower than in TNs but were still higher than in HCs (**Figure 1B**). In TNs, the frequencies of PD-1^+^CD8^+^ T cells were negatively correlated with CD4^+^ T-cell counts (**Figure 1C** left hand panel, r = 0.4238; p = 0.0064) and the CD4/CD8 ratio (**Figure 1C** middle panel, r = 0.3931; p = 0.0121). Similarly, the frequencies of CD39^+^CD8^+^ T cells in TNs were negatively correlated with CD4^+^ T-cell counts (**Figure 1D** left hand panel; r = 0.3838; p = 0.0145) and the CD4/CD8 ratio (**Figure 1D** middle panel, r = 0.3279; p = 0.0389), and positively correlated with the viral load (**Figure 1D** right hand panel, r = 0.4578; p = 0.003). Overall, PD-1^+^CD39^+^CD8^+^ T cells showed the strongest correlations with the disease progression associated markers (**Figure 1E** left hand panel, the CD4^+^ T-cell counts, r = 0.4970, p = 0.0011; middle panel, the CD4/CD8 ratio, r = 0.3657, p = 0.0203; right hand panel, the viral load, r = 0.3906, p = 0.0127) among the three subsets in TNs. Moreover, we also performed correlation analysis of the frequencies of CD8^+^ T-cell subsets with CD4^+^ T-cell counts, the CD4/CD8 ratio and levels of HIV DNA in ARTs (**Supplementary Figures 1A–C**). A significant negative correlation was observed between the frequencies of PD-1^+^CD39^+^CD8^+^ T cells and HIV DNA (r = -0.6273, p = 0.044) in ARTs (**Supplementary Figure 1C**).

### CD39^+^CD8^+^ T Cells Express High Levels of A2A Receptors, TOX, and Are Sensitive to CADO-Induced Functional Suppression

**Figure 2:**
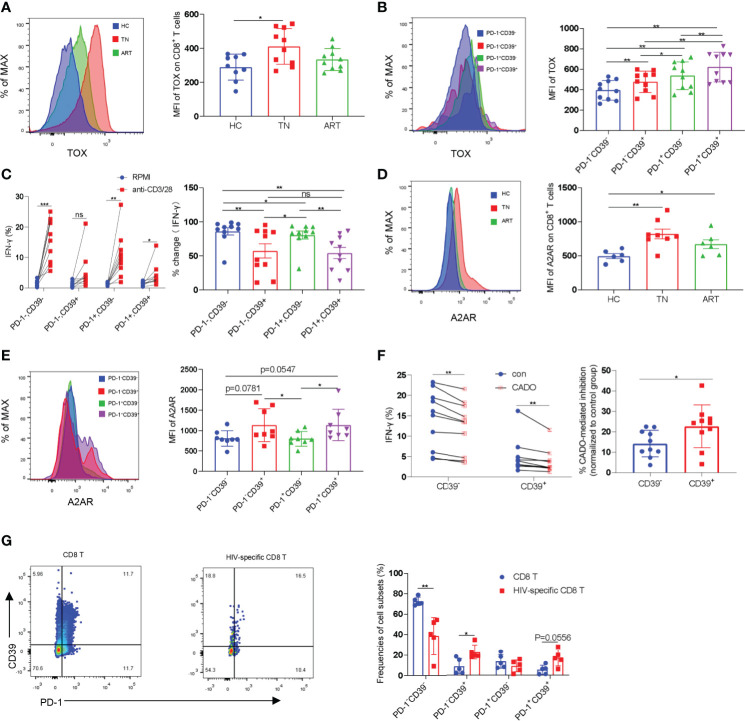
CD39^+^CD8^+^ T cells are highly exhausted and sensitive to CADO-mediated functional inhibition. **(A)** The expression levels of TOX in CD8^+^ T cells from HCs, TNs, and ARTs. **(B)** The expression levels of TOX in PD-1^-^CD39^-^, PD-1^-^CD39^+^, PD-1^+^CD39^-^ and PD-1^+^CD39^+^ CD8^+^ T cells from TNs. **(C)** PBMCs from TNs were stimulated with or without anti-CD3 and anti-CD28 monoclonal antibodies for 6 h. The fold changes in secretion of IFN-*γ* by PD-1^-^CD39^-^, PD-1^-^CD39^+^, PD-1^+^CD39^-^ and PD-1^+^CD39^+^ CD8^+^ T cells were assessed by flow cytometry. % fold change = (anti-CD3/28 treatment group-RPMI group)/anti-CD3/28 treatment group. **(D)** The expression levels of the A2A receptor on CD8^+^ T cells from HCs, TNs, and ARTs. **(E)** The expression levels of the A2A receptor in PD-1^-^CD39^-^, PD-1^-^CD39^+^, PD-1^+^CD39^-^ and PD-1^+^CD39^+^ CD8^+^ T cells from TNs. **(F)** PBMCs from TNs were stimulated with anti-CD3 and anti-CD28 monoclonal antibodies for 6 h with or without CADO. The expressional levels of IFN-*γ* by CD39^-^ and CD39^+^ CD8^+^ T cells were assessed by flow cytometry. The sensitivity to CADO medicated inhibition was compared between CD39^-^ and CD39^+^ CD8^+^ T cells by calculating fold changes. (% CADO-mediated inhibition = (CADO treatment group – anti-CD3/28 treatment group)/anti-CD3/28 treatment group). **(G)** Representative flow cytometry data for CD39 and PD-1 staining in total CD8^+^ T and HIV-specific CD8^+^ T cells of TNs. The frequencies of PD-1^-^CD39^-^, PD-1^-^CD39^+^, PD-1^+^CD39^-^ and PD-1^+^CD39^+^ CD8^+^ T cells in total and HIV-specific CD8^+^ T cells were assessed by flow cytometry. For statistical analyses, a Mann-Whitney U-test or a Wilcoxon matched-pairs signed rank test was performed, *p < 0.05, **p < 0.01, ***p < 0.001, ns, not significant.

The exhausted CD8^+^ T cells found in tumors and chronic infectious diseases are characterized by high levels of expression of inhibitory receptors such as PD-1 and extensive transcriptional changes. It has been demonstrated that the transcription factor TOX is a major regulator in depleted effector cells and that removal of its DNA-binding structural domain reduces PD-1 expression and increases cytokine production (32, 33). We analyzed the expression levels of PD-1 and TOX in CD8^+^ T cells in HCs, TNs, and ARTs. Compared with HCs, the expression levels of TOX in CD8^+^ T cells were significantly increased in TNs. In ARTs, the expression levels of TOX were lower, but were still higher than in HCs (**Figure 2A**). Similar to TOX, the expression levels of PD-1 in CD8^+^ T cells were higher in TNs than in HCs, but were significantly decreased in ART patients compared to TNs (**Supplementary Figure 2A**). Further analysis revealed that in TNs, CD39^+^CD8^+^ T cells expressed higher TOX and PD-1 levels than CD39^-^CD8^+^ T cells (**Figure 2B** and **Supplementary Figure 2B**). To better understand the functional heterogeneity of the CD8**^+^** T-cell subset defined by CD39 and PD-1 expression, we next compared their responses to TCR stimulation *in vitro*. We found that the CD39^-^CD8^+^ T-cell subset had significantly higher levels of IFN-γ secretion, following stimulation by anti-CD3 and anti-CD28 monoclonal antibodies compared to the CD39^+^CD8^+^ T-cell subset (**Figure 2C**). Among the four types of adenosine receptors namely A1R, A2AR, A2BR, and A3R, A2AR is highly expressed in T cells and is responsible for the direct immunosuppressive effects of adenosine (34). Accordingly, we analyzed the expression levels of A2AR in peripheral blood CD8^+^ T cells isolated from HCs, TNs, and ARTs. The expression levels of A2AR in CD8^+^ T cells from TNs were significantly increased compared to HCs. In ARTs, the expression levels of A2AR were lower than in TNs but were still higher than in HCs (**Figure 2D**). Overall, CD39^+^CD8^+^ T cells expressed higher levels of the A2A receptor than CD39^-^CD8^+^ T cells (**Figure 2E**). Moreover, the CD39^+^CD8^+^ T cells were more sensitive to 2-chloroadenosine (CADO)-induced inhibition of IFN-γ secretion than their CD39^-^ counterparts (**Figure 2F**).

Previous studies have shown that CD39^+^CD8^+^ T cells are enriched for antigen-specific cells in HIV-infected and cancer patients (28, 29). We then compared the frequencies of HIV-specific cells in CD8^+^ T cell subsets defined by CD39 and PD-1 using pentamer staining. In consistency with previous studies, HIV-specific CD8^+^ T-cells were enriched in two CD39^+^ subpopulations (**Supplementary Figure 2C** and **Figure 2G**).

### Combined Targeting of Adenosine and PD-1 Signaling Pathways Rescues CD8^+^ T-Cell Function

**Figure 3:**
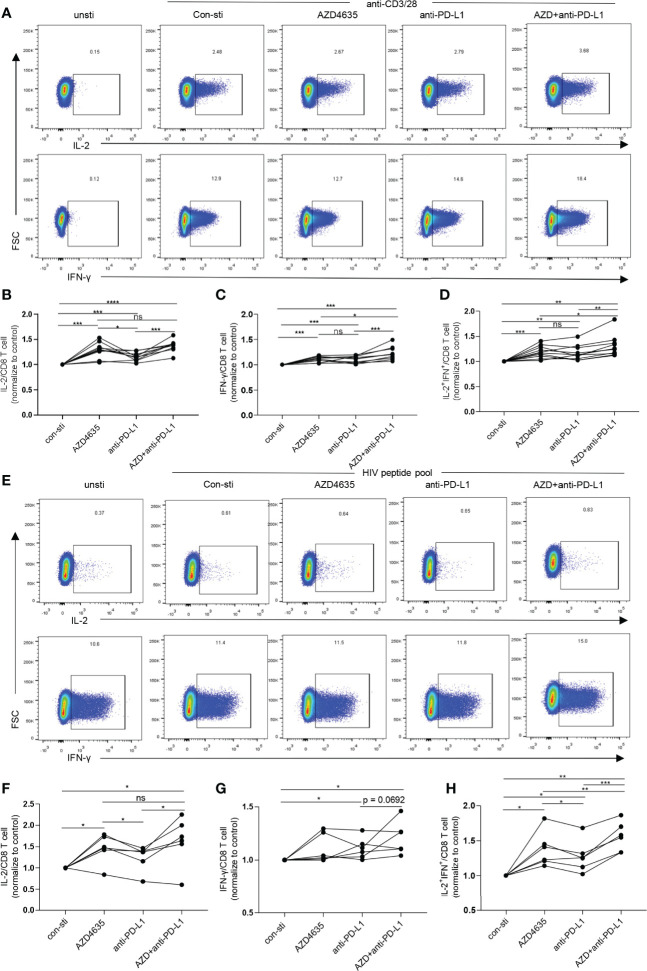
Combined inhibition of the adenosine and PD-1 pathways enhances CD8^+^ T cell function. **(A–D)** PBMCs from TNs were cultured in RPMI 1640 containing 10% fetal bovine serum and stimulated with anti-CD3 (1 ng/mL) and anti-CD28 (1 ng/mL) monoclonal antibodies for 6 h with or without AZD4635 (0.2 µM) or anti-PD-L1 antibody (10 µg/mL), as indicated. The frequencies of IL-2^+^, IFN-γ^+^ and IL-2^+^IFN-γ^+^ CD8^+^ T cells were analyzed by flow cytometry. Representative flow cytometry data are shown in **(A)** and the normalized levels of intracellular IL-2 **(B)**, IFN-γ **(C)** and IL-2 plus IFN-γ **(D)** CD8^+^ T cells were statistically analyzed. **(E–H)** PBMCs from TNs were cultured for 6 h in RPMI 1640 containing 10% fetal bovine serum and overlapping peptides covering the HIV-1 pol, gag, and env antigens (1 μg/mL), CD49d, and anti-CD28. In addition, the cells were treated with AZD4635 (0.2 µM) or anti-PD-L1 antibody (10 µg/mL), as indicated. The secretion of IL-2 and IFN-γ by the CD8^+^ T cell populations were analyzed by flow cytometry. Representative flow cytometry data are shown in **(E)** and the normalize levels of intracellular IL-2 **(F)**, IFN-γ **(G)** and IL-2 plus IFN-γ **(H)** in the CD8^+^ T cells were statistically analyzed. Statistical tests were performed using a paired *t*-test, *p < 0.05, **p < 0.01, ***p < 0.001, ****p < 0.0001, ns, not significant.

Blockade of the PD-1 pathway restores the ability of exhausted CD8^+^ T cells to proliferate, secrete cytokines, and exert their cytotoxic function, resulting in a decrease in viral load during HIV-1/SIV infections (11, 12, 35–37). Our data suggested that the elevated levels of A2A receptor expression in CD8^+^ T cells in TNs are accompanied by reduced CD8^+^ T-cell function (**Figures 2C–F**). Thus, we investigated the effect of targeting both the adenosine and the PD-1 pathways on CD8^+^ T-cell function. Following stimulation with anti-CD3 and anti-28 monoclonal antibodies, the frequencies of IL-2 and IFN-*γ* secreting cells were significantly increased after treatment with anti-PD-L1 antibody and/or AZD4635, a potent and selective A2AR inhibitor (38) (**Figure 3A**). Impressively, combined blockade showed an enhanced effect on reinvigorating CD8 T-cell function than treatment alone with the anti-PD-L1 antibody or AZD4635 (**Figures 3B–D** and **Supplementary Figure 3A**). To assess if blockade of the PD-1 and CD39/adenosine pathway could restore the function of HIV-specific CD8^+^ T cells, we stimulated cells with overlapping peptides covering HIV (pol, gag, and env) antigens. Similarly, we found that blocking the CD39/adenosine and PD-1 pathways restored the capacity of CD8^+^ T cells to produce cytokines (**Figures 3E–H** and **Supplementary Figure 3B**), and in particular additive effects of the AZD4635 and anti-PD-L1 antibody were observed to induce IL-2 (**Figure 3F**) and IL-2 plus IFN-γ (**Figure 3H**) secretion.

### Combined Targeting of the Adenosine and PD-1 Signaling Pathways Promotes CD8^+^ T-Cell-Mediated Inhibition of HIV *In Vitro*

**Figure 4:**
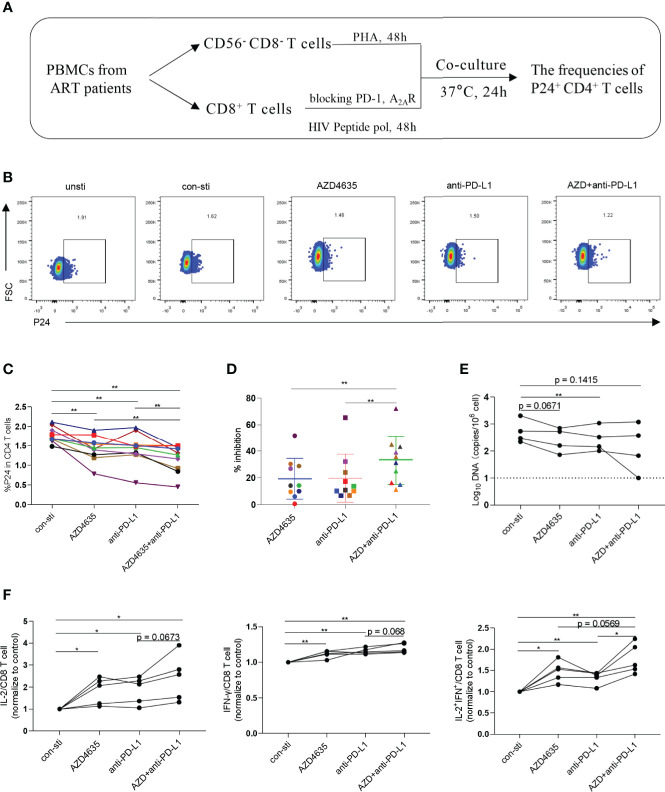
Combined targeting of the adenosine and PD-1 pathways promotes CD8^+^ T-cell-mediated viral inhibition. **(A)** Schematic of *ex vivo* killing assay. **(B)** Representative flow cytometry data showing p24 expression in CD4^+^ T cells after the different treatments with PBMCs from ARTs. **(C)** The frequencies of p24^+^CD4^+^ T cells after the different treatments. **(D)** The inhibitory efficiencies were calculated in the different treatment groups. % inhibition = (Control group - Treatment group)/Control group. **(E)** HIV DNA levels after the different treatments with PBMCs from ARTs. **(F)** The secretion of IL-2, IFN-γ and IL-2 plus IFN-γ by the CD8^+^ T cell populations were analyzed by flow cytometry. Representative flow cytometry data are shown in **(F)** and the normalize levels of intracellular IL-2 (left), IFN-γ (middle) and IL-2 plus IFN-γ (right) in CD8^+^ T cells were statistically analyzed. Statistical tests were performed using a paired *t*-test, *p < 0.05, **p < 0.01.

To investigate whether targeting the PD-1 and/or adenosine pathways could promote CD8^+^ T-cell-mediated inhibition of HIV *in vitro*, we performed an *ex vivo* viral suppression assay. The flow-chart for the experiment is shown in **Figure 4A**. Compared with the control group, the frequency of p24^+^ in CD4^+^ T cells decreased in the presence of an anti-PD-L1 antibody and/or AZD4635 (**Figures 4B, C** and **Supplementary Figure 4A**). Moreover, we observed that the frequency of p24^+^ in CD4^+^ T cells decreased more significantly in the combined blockade treatment group (**Figures 4C, D**). In addition, we measured the levels of HIV DNA among CD4^+^ T cells in different treatment groups after co-cultured with CD8^+^ T cells, with a similar tendency to p24 staining (**Figure 4E**). Meanwhile, the frequencies of IL-2^+^, IFN-*γ*^+^, and IL-2^+^IFN-*γ*^+^ CD8^+^ T cell subsets were increased after treatment with anti-PD-L1 antibody and/or AZD4635, with combined treatment group being the most significant (**Figure 4F** and **Supplementary Figure 4B**). These results indicate that targeting both the CD39/adenosine and the PD-1 pathways can further enhance the antiviral function of CD8^+^ T cells, as compared to targeting only one immune checkpoint pathway.

## Discussion

CD8^+^ T-cell exhaustion is a hallmark of chronic viral infections, and reversal of such dysfunction by ICIs represents a promising therapeutic strategy. PD-1 and CD39 are two well-characterized exhaustion markers and can be co-expressed on CD8^+^ T cells in HIV-1 infected patients (23, 37, 39, 40). In this study, we found that the frequency of PD-1^+^CD39^+^CD8^+^ T cells increased significantly in PLWH and was significantly correlated with parameters reflecting disease progression in TNs. Consistent with previously published studies, our results show that the CD39^+^CD8^+^ T cells express high levels of PD-1 and TOX and are highly sensitive to adenosine-mediated inhibition. Moreover, blocking both the CD39/adenosine and the PD-1 pathways synergistically restored CD8^+^ T-cell function *in vitro* in PBMCs from ART patients. This study therefore, provides a new synergistic strategy to renew CD8^+^ T-cell responses in PLWH.

Targeted immune checkpoint therapy has been widely used to bring revolutionary changes in the treatment of cancer. With respect to PLWH, ICI therapy is an approach that is being explored as an HIV cure due to the potential to activate the latent reservoir and to enhance the immune clearance of infected cells. On the one hand, blockade of PD-1 improves the function of antiviral CD8^+^ T cells during chronic HIV/SIV infections (6, 11, 13). On the other hand, CD4^+^ T cells expressing immune checkpoint markers are enriched in latent HIV-1 (41–43), which might suggest that PD-1 is important in establishing HIV latency (44). Moreover, PD-1 blockade has shown a synergistic effect with other latency reversing agents in several pre-clinical studies (8, 13). However, similar to the low response rate observed for PD-1 blockade in patients with cancer, only one study confirmed that anti-PD-1 therapy appears to be useful in PLWH with cancer (45), necessitating efforts that are aimed at improving efficacy using combined regimens. This strategy was corroborated by a recent report which showed that reversal of HIV latency was achieved by blockade of multiple checkpoint receptors, PD-1, CTLA4, TIM3, and TIGIT without T cell stimulation, and this effect was significantly greater than that induced by latency reversal agents such as vorinostat and bryostatin (46).

Blockade of the CD39/adenosine pathway is an ideal combination with anti-PD-1 therapy in PLWH for several reasons. First, the immunosuppressive properties of adenosine towards immune cells are well documented (47). Signaling through adenosine receptors can modulate both CD4^+^ and CD8^+^ T-cell effector functions, and disrupting this pathway with an anti-CD39 antibody or A2AR inhibitors can reverse the restraints on T cells caused by adenosine. Second, CD39 is abundantly expressed on the surface of Treg cells which are not only susceptible to HIV-1 infection (48, 49) but also exert regulatory functions on other cells such as HIV-specific T cells. In addition, it has been reported that intrinsic adenosine signaling in Tregs is related to HIV reservoir persistence in HIV-1-infected humanized mice (50). Thus, it is likely that targeting the CD39/adenosine pathway, especially in combination with PD-1 pathway blockade, would be beneficial in PLWH. Third, as shown in this study, CD39^+^PD-1^+^CD8^+^ T cells are sensitive to both anti-PD-L1 antibody and A2AR inhibitors. In summary, these findings underscore that inhibiting the CD39/adenosine pathway represents a non-overlapping strategy with PD-1 blockade, and supports the potential clinical efficacy of a combination of both to address the problem of HIV latency.

The combination of suppressing the CD39/adenosine pathway with PD-1 blockade is supported by several pre-clinical studies in cancer research (51, 52) as well as in a clinical proof of concept study (53). However, our research is limited by the cross-sectional design. Further studies with an expanded sample size and animal experiments are needed. Since numerous therapeutic combination therapies, including targeting the CD39/adenosine pathway, are being explored in cancer patients, it is hoped that targeting the CD39/adenosine pathway will improve the treatment effects of ART in PLWH.

## Data Availability Statement

The original contributions presented in the study are included in the article/**Supplementary Material**, further inquiries can be directed to the corresponding author/s.

## Ethics Statement

The studies involving human participants were reviewed and approved by The Fifth Medical Center of Chinese PLA General Hospital. The patients/participants provided their written informed consent to participate in this study.

## Author Contributions

F-SW and CZ conceived the study, wrote the manuscript, and constructed the figures with JL and H-HH. The clinical samples and data were contributed by H-HH and BT. F-SW and CZ revised the manuscript and figures. C-BZ and J-HY performed flow cytometry. TY provided support for the experiment to quantify viral RNA. J-WS, XF, Y-MJ, R-NX, J-YZ, C-BZ, and MS edited the manuscript and provided comments and feedback. M-JZ,WH, Y-LF, and X-YL performed the laboratory work. All authors read and approved the final manuscript.

## Funding

This work was supported by the Innovation Groups of the National Natural Science Foundation of China (81721002) and National Natural Science Foundation of China (grant no. 81772185 and 81901617).

## Conflict of Interest

The authors declare that the research was conducted in the absence of any commercial or financial relationships that could be construed as a potential conflict of interest.
