# Supplementary material for: Reversal of the CD8+ T-Cell Exhaustion Induced by Chronic HIV-1 Infection Through Combined Blockade of the Adenosine and PD-1 Pathways
Source: Front Immunol. 2021 Jun 10;12:687296. doi: 10.3389/fimmu.2021.687296 (PMC8222537; doi:10.3389/fimmu.2021.687296)
Supplement: Supplementary file 1 [file DataSheet_1.pdf]

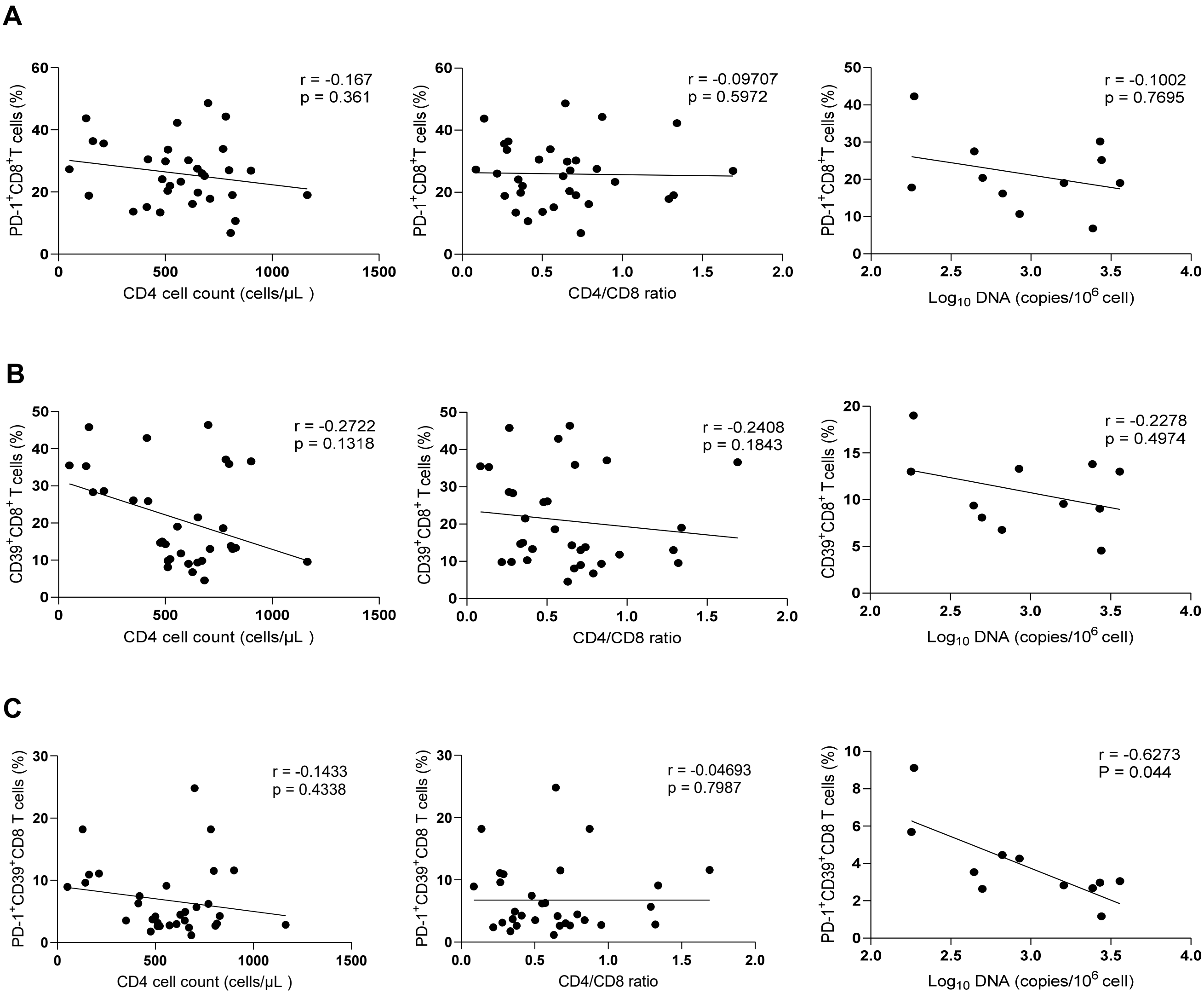

Supplementary Figure 1. Frequencies of PD-1$^+$CD8$^+$, CD39$^+$CD8$^+$, and PD-1$^+$CD39$^+$CD8$^+$ T cells and their correlations with CD4$^+$ T-cell counts, the CD4/CD8 ratio, and viral load in ARTs. **(A-C)** Correlations between the frequencies of PD-1$^+$CD8$^+$ (**A**), CD39$^+$CD8$^+$ (**B**) and PD-1$^+$CD39$^+$ CD8$^+$ (**C**) T cells with CD4$^+$ T-cell counts, the CD4/CD8 ratio, and HIV DNA in ARTs. Each dot represents one individual. For the statistical analyses, a Mann–Whitney U-test was performed. Correlations were performed using a Spearman rank correlation test. Solid line, linear growth trend; r, correlation coefficient.

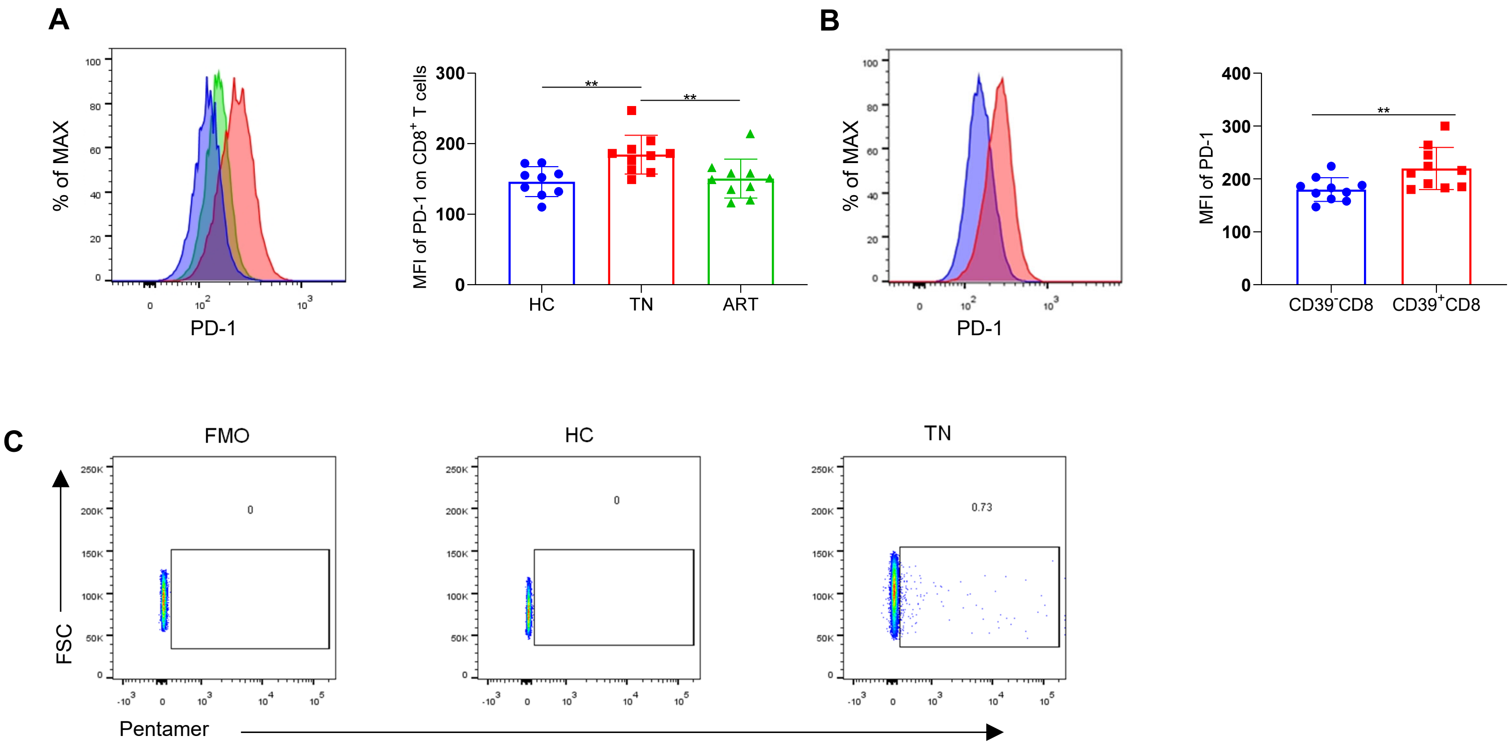

Supplementary Figure 2. High expression of PD-1 in $CD39^{+}CD8^{+}$ T cells of HIV-1-infected patients.

(A) The expression levels of PD-1 in $CD8^{+}$ T-cells from HCs, TNs, and ARTs. **(B)** The expression levels of PD-1 in $CD39^{-}CD8^{+}$ and $CD39^{+}CD8^{+}$ T cells from TNs. **(C)** Representative flow cytometry data of FMO controls, pentamer on total $CD8^{+}$ T cells from HCs and TNs. For statistical analyses, a Mann-Whitney U-test or a Wilcoxon matched-pairs signed rank test was performed, $**p < 0.01$.

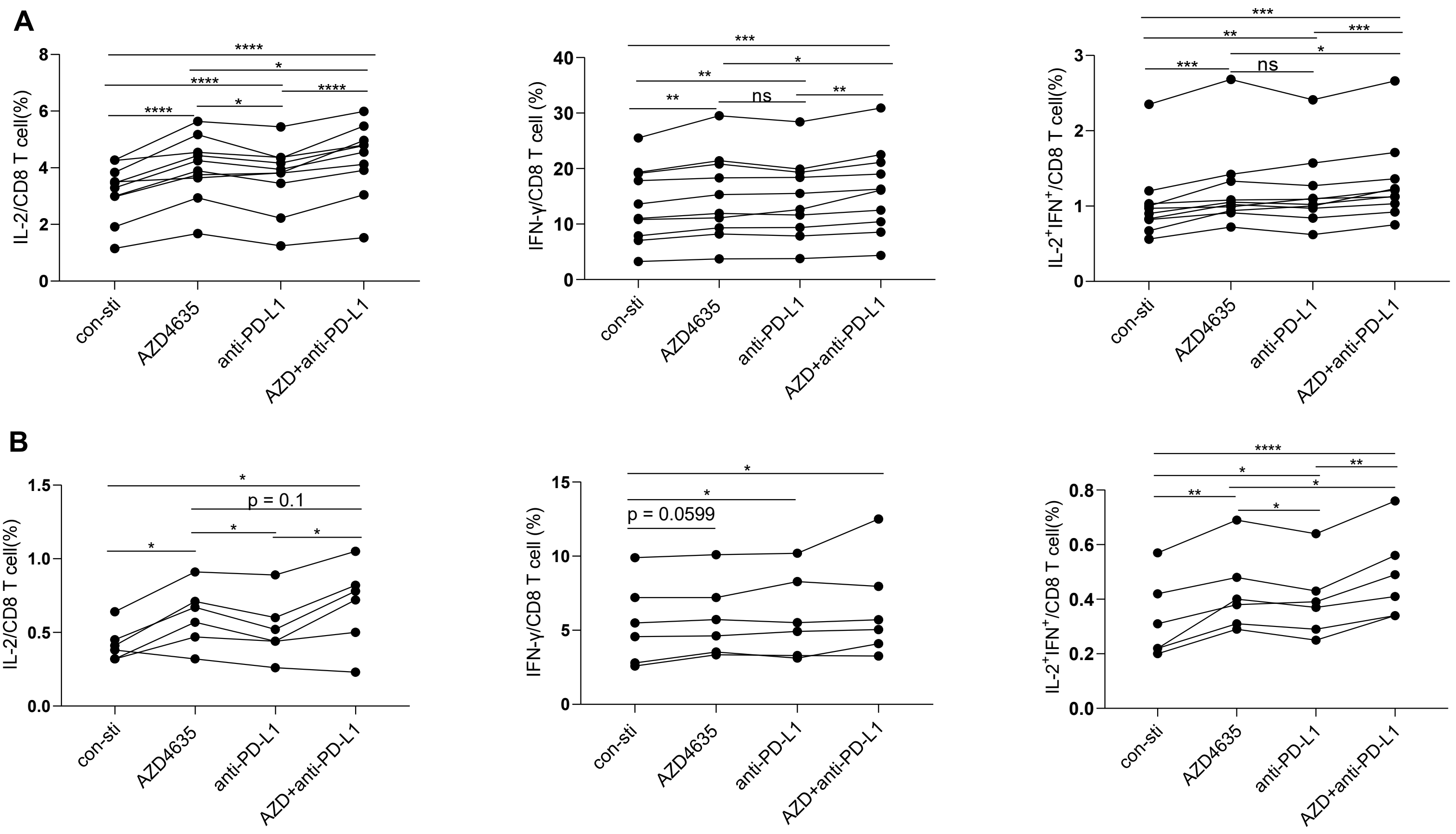

Supplementary Figure 3. Combined inhibition of the adenosine and PD-1 pathways enhances CD8+ T cell function and HIV-specific CD8+ T cell function.

(A) The unnormalized data for Figure 3B-D. (B) The unnormalized data for Figure 3F-H. Statistical tests were performed using a paired *t*-test, *p < 0.05, **p < 0.01, ***p < 0.001, ****p < 0.0001, ns, not significant.

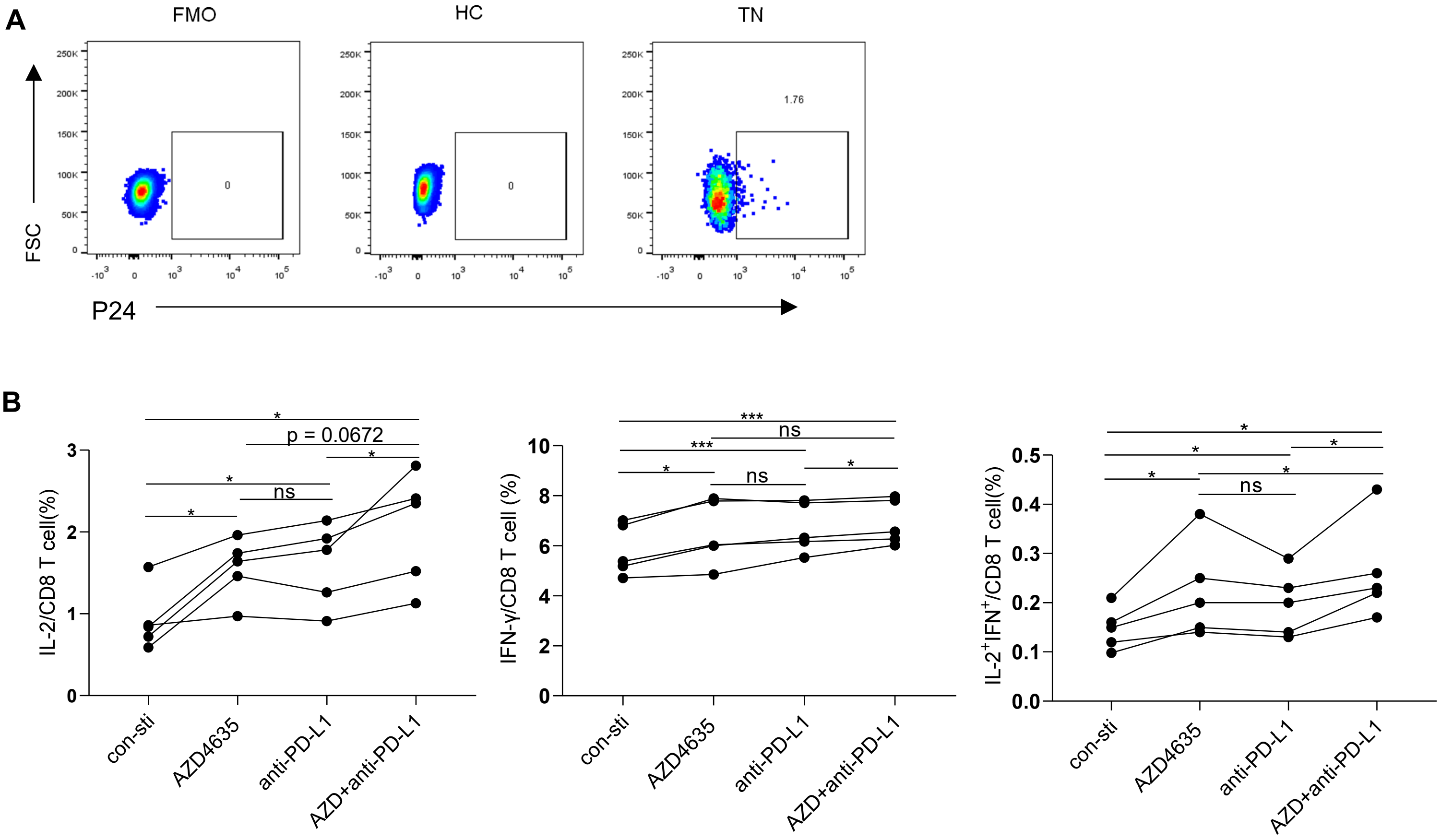

Supplementary Figure 4. Combined targeting of the adenosine and PD-1 pathways promotes $CD8^+$ T-cell-mediated viral inhibition.

(A) Representative flow cytometry data of FMO controls, p24 in $CD4^+$ T cells from HCs and TNs. **(B)** The unnormalized data for Figure 4F. Statistical tests were performed using a paired *t*-test, *$p < 0.05$, ***$p < 0.001$, ns, not significant.
